# Learning outcomes of a flipped classroom teaching approach in an adult-health nursing course: a quasi-experimental study

**DOI:** 10.1186/s12909-020-02240-z

**Published:** 2020-09-18

**Authors:** Jun-Yu Fan, Ying-Jung Tseng, Li-Fen Chao, Shiah-Lian Chen, Sui-Whi Jane

**Affiliations:** 1 Department of Nursing & Graduate Institute of Nursing, Chang Gung University of Science and Technology, Division of Nursing, Chang Gung Memorial Hospital, Linkou Branch, 261, Wen-Hua 1st Road, Kwei-Shan, Tao-Yuan, 33303 Taiwan (R.O.C.); 2 Department of Nursing, Chang Gung University of Science and Technology, 261, Wen-Hua 1st Road, Kwei-Shan, Tao-Yuan, 33303 Taiwan (R.O.C.); 3 Department of Nursing, National Taichung University of Science and Technology, No.129, Sec. 3, Sanmin Rd., North Dist., Taichung City, 40401 Taiwan (R.O.C.); 4 Department of Nursing & Graduate Institute of Nursing, Chang Gung University of Science and Technology, 261, Wen-Hua 1st Road, Kwei-Shan, Tao-Yuan, 33303 Taiwan (R.O.C.)

**Keywords:** Flipped classroom, Adult-health nursing, Bachelor of science in nursing

## Abstract

**Background:**

New teaching strategies must be developed not only to enhance nurse’s competence but also to allow nurses to respond to the complex health care needs of today’s society. The purpose of this study was to explore the learning outcomes of a flipped classroom teaching approach in an adult-health nursing course for students in a two-year Bachelor of Science in Nursing program.

**Methods:**

The study had a quasi-experimental design. An 18-week flipped classroom teaching approach was applied in an adult-health nursing course. In total, 485 nursing students enrolled in the study, with 287 in the experimental group and 198 in the control group. The Self-Evaluated Core Competencies Scale, Metacognitive Inventory for Nursing Students, Self-Directed Learning Readiness Scale, and self-designed learning satisfaction questionnaire were used to evaluate the students’ learning outcomes.

**Results:**

The experimental group showed a statistically significant increase in the overall scores for self-evaluated core competencies, the “self-modification” subscale of the Metacognitive Inventory for Nursing Students, and in overall self-directed learning readiness; further, they also showed high levels of course satisfaction.

**Conclusions:**

A flipped classroom teaching approach had a positive impact on student’s learning motivation and contributed to better learning outcomes in an adult-health nursing course. The flipped classroom combined with hybrid teaching methods is a suitable and effective learning strategy for a registered nurse (RN) to Bachelor of Science in Nursing (BSN) program to tackle today’s complex revolution in nursing curricula, and may enhance nursing students’ abilities to address numerous challenges.

## Background

Health professional education is expected to produce graduates who are proficient in core competencies and have the ability to provide safe, high-quality, patient-centered care [1]. However, many employers find that recent nursing graduates are not competent enough at performing basic clinical tasks [2, 3]. Thus, new approaches and educational models must be developed to allow nurses to respond rapidly to the changes in the medical field [1]. Past educational methods (e.g., teacher-centered lecturing) for nurses are no longer adequate for addressing the complex health care needs of today’s society [4, 5]. Therefore, many nursing schools have become aware of this transition and begun to review their missions, core competencies, and competency indicators, while also initiating a shift from training students in task-based proficiencies to providing education in higher-level competencies, such as decision-making, quality improvement, systems thinking, evidence-based practice, and inter-professional teamwork and collaboration [1].

### Two-year RN-to-BSN program in Taiwan

The universal goal of educating nursing professionals is to produce graduates who can meet patients’ needs and deliver safe, quality patient care. In Taiwan, to become a registered nurse (RN), students must graduate from an accredited program. The two options available are an associate degree or a bachelor’s degree in nursing. The Associate Degree in Nursing (ADN) is a five-year program which is offered by junior colleges and admits graduates from junior high schools. The Bachelor of Science in Nursing (BSN) degree program, which is offered through the university educational system (including universities, colleges, universities of technology, and technical colleges), is usually 4 years in duration and requires 12 years of prior education [6]. Both BSN and ADN graduates must pass professional and technical personnel examinations before becoming RNs. These examinations are administered by the Examination Yuan. In Taiwan, an RN with an ADN can provide the same level of care as an RN with a BSN. Regarding long-term career mobility, however, nurses with BSNs tend to have more options for professional development and advancement (such as administrative and leadership positions or various nursing specialties), skill-building, and cultural awareness, as well as obtain higher salaries [7].

To help further ADN nurses’ education, an RN-to-BSN program was developed, and by Fall 2019, there were 17 accredited RN-to-BSN programs offered by public or private universities, colleges, universities of technology, or technical colleges throughout Taiwan. All the RN-to-BSN programs are classroom-based, and off-campus online programs are not permitted by the Ministry of Education.

Our university is a private university with two campuses, 235 km apart: the Linkou (L) campus in northern Taiwan and the Chiayi (C) campus in southern Taiwan, with a total of approximately 6400 students, as of 2020. Our RN-to-BSN program is a two-year program, offering a total of 72 credits, with an average of 18 credits per semester, including 450 h of hospital-based clinical practicum. There are approximately 300 and 200 students per year in L and C campuses, respectively. Both campuses share the same course list and conduct the same core courses simultaneously. The faculty members from both campuses shared input in terms of course design, revision, and evaluation via an internet meeting before course start and after course end, but faculty members and students do not cross campus due to distance inconvenience. Our curriculum was designed to help ADN nurses reach advanced levels of competencies in critical thinking and analysis, evidence-based practice, problem-solving skills, communication and informatics, decision-making and clinical judgment, teamwork and collaboration, and life-long self-directed learning. To meet our desired learning outcomes, we have applied various new teaching strategies, such as scenario simulation, problem-based learning (PBL), team-based learning (TBL), blended learning (BL), and objective simulation clinical examination to equip students with the competencies and skills needed to deliver quality care in today’s complex health care system.

### Flipped classrooms and hybrid teaching methods

A BL method known as the “flipped classroom” approach is rapidly growing in popularity in health care educational disciplines with the purpose of activating or facilitating students’ engagement in the learning process [8–10]. Many approaches such as PBL, TBL, simulated-based learning, role play, or web-based learning are used to flip students’ learning style from teacher-centered (passive) to learner-centered (active), as well as to increase student engagement, enhance the development of critical thinking, and improve learning outcomes [8, 11–13]. The key elements of the flipped classroom include pre-class content, in-class activities, post-class assessment, and student-triggered inquiry [8, 14, 15].

To create a successful flipped classroom, students’ intrinsic motivations are a key element in achieving desired learning outcomes. In the digital era, instructors or teachers often combine computer-mediated technology and face-to-face in-class activities to enhance students’ engagement in pre-class work, in-class learning, and post-class assignments, as well as to help students achieve their goals during the flipped classroom processing period [8, 12, 13]. Several studies have shown that students prefer a hybrid course structure and their learning outcomes can improve in knowledge, decision-making skills, satisfaction, thinking abilities, and reflections concerning clinical practice [16–26]. Most of these studies also demonstrated the mastery learning behavior suggested by Bloom [27], which posits that learning activities applied in class, such as professional knowledge, relevant skills, and scholarly inquiry, might transfer to real clinical practice [8].

The flipped classroom combined with a hybrid teaching course structure provides students with not only a flexible way to learn materials (mostly online) at their own pace before class (autonomy) but also reinforces students’ in-class discussion with peers (relatedness), and reevaluates students’ strengths and weaknesses (competence). Ultimately, the flipped classroom may help students to develop self-directed learning skills and cultivate life-long learning habits [20]. Thus, the purpose of the present study was to explore students’ learning outcomes of a flipped classroom teaching approach in adult-health nursing course in a two-year RN-to-BSN program.

## Methods

### Research design and samples

This study was a quasi-experimental design and was conducted at a private university with two campuses as previously described in the “Two-year RN-to-BSN program in Taiwan” section of this paper. The study was conducted from September 2015 to February 2016. There were 504 total students enrolled in the two-year RN-to-BSN program with 304 and 200 students at the L and C campuses, respectively. All 504 RN-to-BSN students were potential participants. To minimize intervention “contamination” between experimental and control participants, the students from L campus belonged to the experimental group (EG) and those from C campus belonged to the control group (CG).

### Flipped classroom intervention

In the present study, a flipped classroom was implemented in an adult-health nursing course on L campus that incorporated face-to-face TBL and simulation activity and online self-directed learning (via the “e-campus” platform). The adult-health nursing course is one of the core nursing courses taught in the ADN program. The reasons why the adult-health nursing course was chosen in our two-year RN-to-BSN program curriculum was because its application and utilization play an important role in clinical practice, and the majority of our graduates’ work in medical or surgical wards. Thus, more in-depth course work from an ADN level is necessary, and this may help students have a better understanding of the cultural, economic, and social issues affecting patients.

Before the flipped classroom was implemented, we held several faculty-training sessions on L campus to ensure consistency in content and teaching materials, including quiz questions used in TBL (in-class), the simulation scenario (in-class), the assignment format (post-class), and reading materials (pre-class). The adult-health nursing course is a 36-h, two-credit course taught during the first semester of the first academic year at both campuses. In terms of the content of the adult-health nursing course, we divided 36 h into five blocks for five topics based on the leading causes of death in Taiwan: diabetes mellitus, chronic obstructive pulmonary disease, acute coronary syndrome, stroke, and cancer. We adapted an in-depth approach to course contents by integrating pathophysiology, physical assessments, nursing care, and psychosocial issues. Each topic was addressed in four phases.

The first phase was an online self-directed learning pre-class phase in which students reviewed assigned reading materials via the online e-campus platform. Two weeks before the course began, the instructor was asked to upload reading materials (e.g., syllabus, related papers or videos, case descriptions for simulation exercises, assignments, and reflection and evaluation forms) on e-campus. The second phase was a face-to-face TBL in-class activity. The two- to four-hour TBL process began with an individual quiz, followed by a group discussion, and ended with an appeal or argument process. The third phase was a face-to-face simulation activity in a laboratory. There was a two- to four-hour simulation exercise related to the TBL content, with a case based on actual clinical practice. The last phase involved completing a post-class assignment on e-campus. The students were asked to submit their completed assignments, reflection reports, and course evaluations, as well as any comments they had concerning the e-campus platform. The e-campus platform also facilitated interaction, discussion, and announcements.

### Traditional teaching

**Fig. 1:**
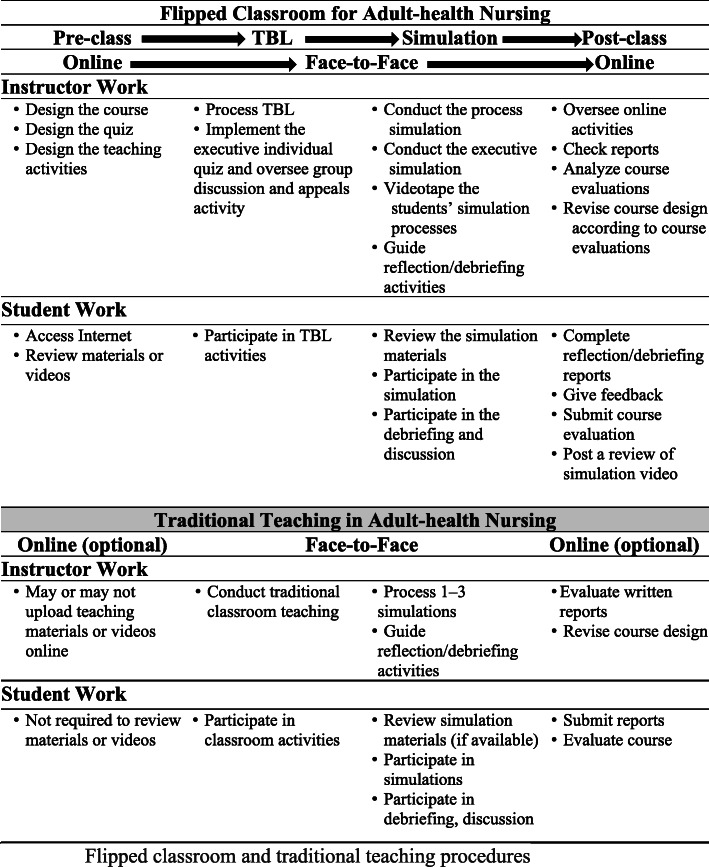
Flipped classroom and traditional teaching procedures

In the first phase, 2 weeks before the course began, it was optional for the instructor to upload adult-health nursing course teaching materials or videos online and the students were not required to review materials or videos before the course began. The second phase was face-to-face traditional classroom teaching during which the instructor delivered the knowledge mostly using slides. The third phase was a face-to-face simulation activity (at least 1 simulation activity). The students participated in the classroom and laboratory activities in both face-to-face phases. The last phase, the instructor competed a written evaluation of the course in the form of a written report and/or revised the course design for next semester. The students were asked to submit homework reports and evaluate the course. Figure 1 shows the flipped classroom and traditional teaching procedures.

### Instruments

Students’ demographic data, including age and gender were collected. To understand the learning outcomes of the flipped classroom, particularly regarding the students’ mental self-evaluation processes (also referred to as metacognitive ability) and self-directed learning skills the following instruments were used [3].

### Self-evaluated Core competencies scale

The Self-Evaluated Core Competencies Scale (SECC) includes eight core competencies stipulated by the Taiwan Nursing Accreditation Council [28]. The SECC contains 55 items, grouped into two sections and eight subscales. The *humanity/responsibility* section includes four subscales: caring (6 items), ethics (9 items), accountability (7 items), and life-long learning (5 items). The c*ognitive/performance* section also includes four subscales: communication and teamwork capability (6 items), critical thinking and reasoning (5 items), general clinical skills (9 items), and basic biomedical science (5 items). An additional three items measure overall competence, confidence conducting clinical practice, and the ability to adapt after graduation. An 8-point Likert scale, ranging from 0 (cannot assess) to 7 (excellent competence), is used to indicate the level of competency. Higher scores indicate higher levels of competency, ranging from 0 to 385 points. The SECC showed a good Cronbach’s alpha of .80. Cronbach’s alphas for the humanity/responsibility section (.81), cognitive/performance sections (.63), and eight subscales (ranging from .63 to .81) all demonstrated good internal consistency as well [28].

### Metacognitive inventory for nursing students

The Metacognitive Inventory for Nursing Students (MINS) developed by Hsu [29] was used to measure the association between participants’ knowledge and their awareness of their own thoughts and behaviors. The MINS includes 28 items and five subscales: self-monitoring (7 items), self-modification (7 items), self-awareness (6 items), effective learning (3 items), and problem solving (5 items). Scores are measured using a 5-point Likert scale ranging from 1 (never) to 5 (always), with higher scores indicating higher metacognitive ability (ranging from 28 to 140). The MINS demonstrated good internal consistency, and Cronbach’s alpha was .94 for the total scale and ranged from .73 to.90 for the five subscales, explaining 53.09% of the variance [29].

### Self-directed learning readiness scale

The Self-Directed Learning Readiness Scale (SDLRS) used in this study was adapted from Tang [30] to examine participants’ readiness to perform self-directed learning. The SDLRS contains 36 items grouped into six subscales: effective learning (6 items), love of learning (7 items), learning motivation (5 items), active learning (9 items), independent learning (5 items), and creative learning (4 items). A 5-point Likert scale ranging from 1 (never) to 5 (most of the time) was used, with higher scores indicating higher trends of self-directed learning (total scores range from 0 to 180 points). Cronbach’s alpha was .92 for the total scale, explaining 54.33% of the variance. For each subscale, Cronbach’s alphas ranged from .70 to .88, for this study.

### Flipped classroom satisfaction questionnaire

A satisfaction questionnaire was developed exclusively for this study. This questionnaire has 35 items grouped into four subscales: teacher’s teaching (14 items), course content (8 items), learning environment (10 items), and administrative service (3 items). The 35 items measuring satisfaction were scored using a scale ranging from 1 (totally disagree) to 5 (totally agree), with higher scores representing higher levels of satisfaction. In current study, the satisfaction questionnaire demonstrated a good internal consistency with Cronbach’s alphas of .98, .95, .94, .95, and .82 for the total scale, teacher’s teaching subscale, course content subscale, learning environment subscale, and administrative service subscale, respectively.

### Procedure

This study was approved by an institutional review board (IRB No. 104-5709C) before data collection. During the first week of the adult-health nursing course, either the principal investigator or co-investigator explained the study’s purpose, as well as the procedures regarding the distribution of the questionnaires, to potential participants. For the entire semester, the EG students on L campus received the flipped classroom method, as shown in Fig. 1, while those in the CG received traditional teaching methods (see Fig. 1). Before (pre-test) and after (post-test) the adult-health nursing course, students in both groups completed the SECC, MINS, and SDLRS questionnaires. The flipped classroom satisfaction questionnaire was administered only to those in the EG.

### Statistics

We used the generalized estimating equation (GEE) model to evaluate the differences between pre- and post-intervention’s scores on the SECC, MINS, and SDLRS scores. Each GEE model included a main effect of group (EG vs. CG), a main effect of time (post-test vs. pre-test), and a two-way interaction effect of group by time. The parameter estimate of the two-way interaction effect indicates group differences concerning the change from the pretest to the post-test. Data analysis was performed using SPSS 22 (IBM SPSS, Armonk, NY: IBM Corp).

## Results

### Participants’ characteristics

A total of 504 nursing students who were in the RN-to-BSN program were our potential participants. Seven students refused to participate, and we ultimately obtained 497 written informed consent forms. Twelve participants were excluded as a result of incomplete data. A total of 485 nursing students participated (mean age 20.18 ± .59), and these individuals were assigned to either the EG (*n* = 287) or the CG (*n* = 198). The majority were female (465; 95.90%). In terms of homogeneity between groups, there were no significant differences in age and gender but the EG scored significantly higher on one subscale (estimated competence after graduation) of the SECC and on two subscales (self-monitoring and self-awareness), as well as on the mean overall score, of the MINS in CG. The overall pre-test and post-test scores, scores for the SECC, MINS, SDLRS, and flipped classroom satisfaction subscales are shown in Table 1.

**Table 1:** Descriptive statistics of each outcome measure in the pre-test and post-test

Variable	CG (*n* = 198)		EG (*n* = 287)	
	Pre-test	Post-test	Pre-test	Post-test
Age (years)	20.24 ± 0.81		20.14 ± 0.37	
Gender (female) n (%)	279 (57.5%)		186 (38.4%)	
SECC				
Basic biomedical science	4.60 ± 0.75	4.86 ± 0.73	4.62 ± 0.69	5.00 ± 0.59
General clinical skills	5.05 ± 0.70	5.21 ± 0.71	5.07 ± 0.68	5.47 ± 0.62
Communication and teamwork capability	5.55 ± 0.73	5.61 ± 0.80	5.58 ± 0.75	5.85 ± 0.73
Critical thinking and reasoning	4.84 ± 0.82	5.12 ± 0.73	4.85 ± 0.80	5.33 ± 0.70
Caring	5.73 ± 0.76	5.68 ± 0.84	5.69 ± 0.79	5.98 ± 0.72
Ethics	5.97 ± 0.73	5.85 ± 0.82	5.90 ± 0.72	6.17 ± 0.66
Accountability	5.78 ± 0.79	5.81 ± 0.82	5.77 ± 0.75	6.05 ± 0.66
Life-long learning	5.40 ± 0.87	5.54 ± 0.84	5.44 ± 0.81	5.72 ± 0.71
Estimated competence after graduation*	4.86 ± 0.97	5.22 ± 0.93	5.03 ± 0.96	5.34 ± 0.81
Overall	5.38 ± 0.66	5.48 ± 0.63	5.39 ± 0.64	5.71 ± 0.56
MINS				
Self-monitoring**	3.20 ± 0.64	3.44 ± 0.59	3.39 ± 0.63	3.55 ± 0.63
Self-modification	3.65 ± 0.58	3.75 ± 0.60	3.65 ± 0.56	3.87 ± 0.57
Self-awareness***	2.93 ± 0.58	3.29 ± 0.60	3.12 ± 0.59	3.30 ± 0.61
Effective learning	3.48 ± 0.56	3.71 ± 0.58	3.50 ± 0.59	3.68 ± 0.60
Problem solving	3.30 ± 0.55	3.47 ± 0.58	3.39 ± 0.57	3.56 ± 0.57
Overall*	3.30 ± 0.49	3.52 ± 0.52	3.41 ± 0.51	3.59 ± 0.52
SDLRS				
Learning motivation	3.47 ± 0.63	3.58 ± 0.63	3.46 ± 0.58	3.65 ± 0.59
Active learning	3.72 ± 0.47	3.73 ± 0.56	3.66 ± 0.51	3.81 ± 0.48
Love of learning	3.48 ± 0.58	3.55 ± 0.56	3.43 ± 0.55	3.61 ± 0.55
Independent learning	3.46 ± 0.44	3.37 ± 0.53	3.39 ± 0.51	3.30 ± 0.58
Creative learning	3.51 ± 0.63	3.57 ± 0.61	3.46 ± 0.62	3.60 ± 0.62
Effective learning	3.04 ± 0.70	3.29 ± 0.68	3.11 ± 0.63	3.31 ± 0.68
Overall	3.45 ± 0.43	3.53 ± 0.46	3.42 ± 0.43	3.57 ± 0.43
Flipped classroom Satisfaction				
Teacher’s teaching***		3.82 ± 0.55		4.13 ± 0.51
Course content***		3.72 ± 0. 61		3.97 ± 0. 63
Learning environment***		3.97 ± 0.61		4.20 ± 0.57
Administration service***		3.73 ± 0.66		4.03 ± 0.61
Overall***		3.83 ± 0.55		4.11 ± 0.47

*CG* Control group, *EG* Experimental group, *SECC* Self-Evaluated Core Competencies Scale, *MINS* Metacognitive Inventory for Nursing Students, *SDLRS* Self-Directed Learning Readiness Scale. *p < 0.05, **p < 0.01, ***p < 0.001. (Independent samples *t*-test was used to identify the statistical significance in the pre-test between the control and experimental groups)

### Evaluation of the differences between pre- and post-intervention scores on the SECC

**Fig. 2:**
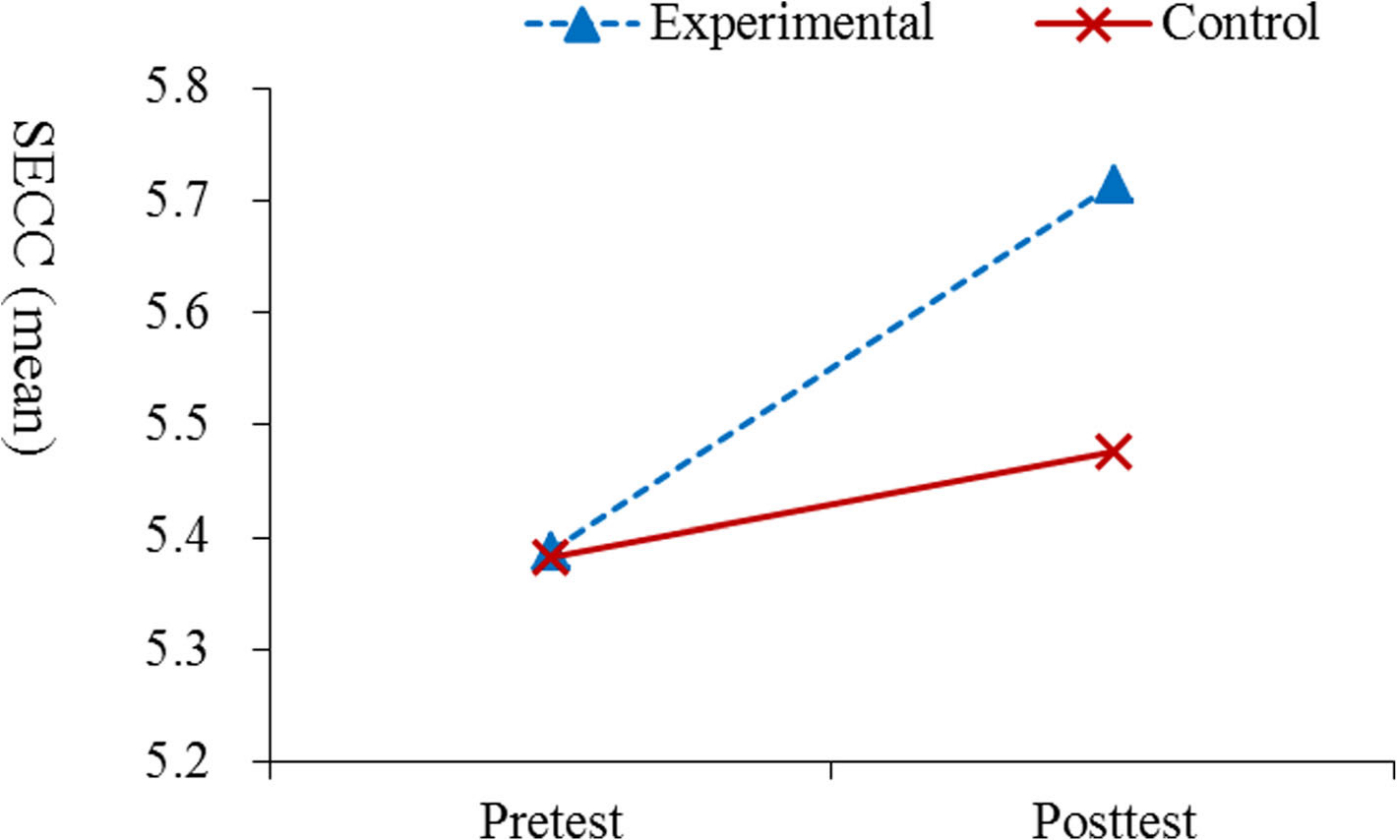
Results of the GEE analysis regarding the pre- and post-intervention on overall SECC

Table 2 summarizes the results of GEE regarding the pre- and post- intervention scores on the SECC. The results showed that, in most subscales, participants in the EG demonstrated greater improvements than those in the CG (*p* < 0.01; Fig. 2); however, group differences regarding changes in basic biomedical science, life-long learning, and estimated competence after graduation were not significant.

**Table 2:** Summary of GEE analysis results regarding the pre- and post-intervention SECC scores

Parameter	Intercept		Group (E vs. C)		Time (Post vs. Pre)		Interaction between groups by time	
Variable	*B*	*P*	*B*	*P*	*B*	*P*	*B*	*P*
Basic biomedical Science	4.33	< .001	−0.10	.393	0.27	< .001	0.12	.054
General clinical skills	4.89	< .001	−0.23	.039	0.16	.001	0.24	< .001
Communication and teamwork capability	5.50	< .001	−0.20	.092	0.05	.323	0.22	.001
Critical thinking and Reasoning	4.55	< .001	−0.17	.192	0.29	< .001	0.19	.009
Caring	5.77	< .001	−0.37	.003	−0.04	.451	0.33	< .001
Ethics	6.09	< .001	−0.46	<.001	−0.12	.024	0.39	< .001
Accountability	5.75	< .001	−0.26	.035	0.03	.579	0.25	< .001
Life-long learning	5.26	< .001	−0.10	.480	0.14	.019	0.14	.073
Estimated competence after graduation	4.49	< .001	0.23	.139	0.37	< .001	−0.06	.526
Overall	5.29	< .001	−0.23	.020	0.09^*^	.023	0.23	< .001

*GEE* Generalized estimating equation, *SECC* Self-Evaluated Core Competencies Scale, *E* Experimental group, *C* Control group; *B* indicates the estimated parameter derived from GEE analysis

### Evaluation of the differences between pre- and post-intervention scores on the MINS

Table 3 lists the results of GEE regarding the pre- and post-intervention MINS scores. Specifically, GEE analyses showed that the improvement from pre-test to post-test in the mean scores of self-modification in the EG were greater than that in the CG (*B* = 0.12, *p* < 0.05). However, the results regarding self-awareness were the opposite; the CG showed greater improvement in self-awareness from the pre-test to the post-test (*B* = − 0.18, *p* < 0.001).

**Table 3:** Summary of GEE analysis results regarding the pre- and post-intervention MINS scores

Parameter	Intercept		Group (E vs. C)		Time (Post vs. Pre)		Interaction between group by time	
Variable	*B*	*P*	*B*	*P*	*B*	*P*	*B*	*P*
Self-monitoring	2.96	<.001	0.27	.010	0.24	<.001	− 0.08	.159
Self-modification	3.54	<.001	−0.11	.225	0.10	.015	0.12	.033
Self-awareness	2.57	<.001	0.37	<.001	0.36	<.001	−0.18	<.001
Effective learning	3.26	<.001	0.05	.584	0.22	<.001	−0.04	.507
Problem solving	3.13	<.001	0.10	.265	0.17	<.001	−0.01	.909
Overall	3.09	<.001	0.14	.058	0.22	<.001	−0.04	.399

*GEE* Generalized estimating equation, *MINS* Metacognitive Inventory for Nursing Students, *E* Experimental group, *C* Control group, B indicates the estimated parameter derived from GEE analysis

### Evaluation of the differences between pre- and post-intervention scores on SDLRS

**Fig. 3:**
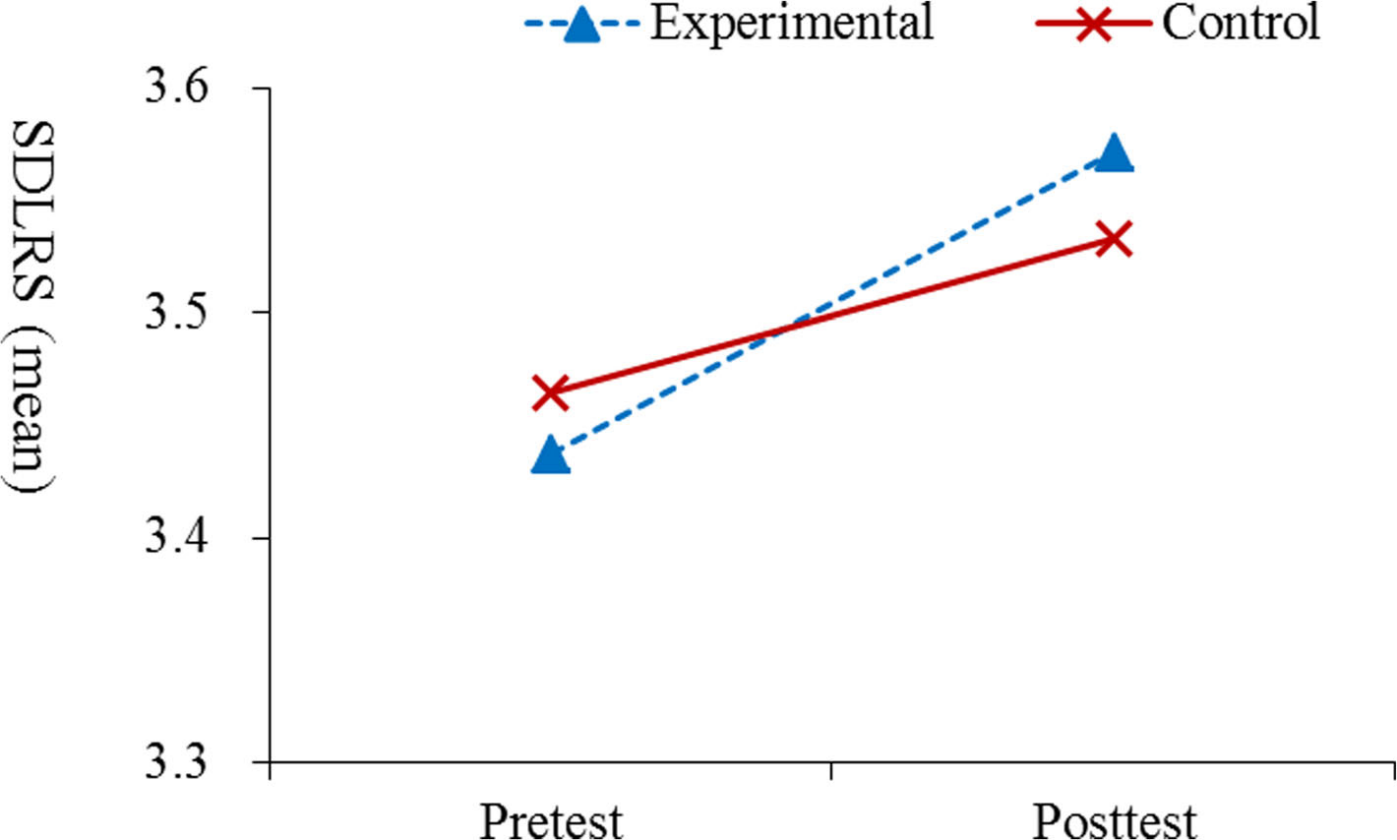
Results of the GEE analysis regarding the pre- and post-intervention on overall SDLRS

Table 4 shows the results of GEE regarding the pre- and post-intervention SDLRS scores (*B* = 0.07, *p* = 0.039; Fig. 3)*.* The intervention was found to improve the mean scores of active learning (*B* = 0.13, *p* < 0.01) and desired learning (*B* = 0.10, *p* < 0.05). However, no significant differences between pre- and post- intervention scores were observed for the other SDLRS subscales.

**Table 4:** Summary of GEE analysis results regarding the regarding the pre- and post-intervention SDLRS scores

Parameter	Intercept		Group (E vs. C)		Time (Post vs. Pre)		Interaction between group by time	
Variable								
	*B*	*P*	*B*	*P*	*B*	*P*	*B*	*P*
Learning motivation	3.36	<.001	−0.10	.246	0.11	.004	0.09	.065
Active learning	3.71	<.001	−0.19	.010	0.01	.688	0.13	.003
Love learning	3.40	<.001	−0.14	.090	0.08	.042	0.10	.032
Independent learning	3.54	<.001	−0.07	.311	−0.09	.014	0.00	.915
Creative learning	3.44	<.001	−0.13	.184	0.06	.154	0.08	.127
Effective learning	2.80	<.001	0.12	.213	0.24	<.001	−0.05	.334
Overall	3.40	<.001	−0.09	.115	0.07	.009	0.07	.039

*GEE* Generalized estimating equation, *SDLRS* Self-Directed Learning Readiness Scale. *E* Experimental group, *C* Control group, B indicates the estimated parameter derived from GEE analysis

### Flipped classroom satisfaction questionnaire

The results showed that the overall level of satisfaction was 3.99 ± 0.50, approaching a score of 4 (agree). The EG demonstrated high satisfaction scores overall and for all four subscales (all *p* values < .001).

## Discussion

### Evaluation of the differences between pre- and post-intervention scores on the SECC

This study showed that our flipped classroom teaching approach has a potential to create positive learning outcomes in terms of clinical skills, communication, and teamwork capabilities, as well as the competencies of critical thinking, caring, work ethic, and accountability. Our findings were partially consistent with those of Jang and Hong [31] (critical thinking), Durmaz et al. [17] (admission skills), Kim and Kim [23] (fundamental nursing practice course), Gerdsprasert et al. [19] (intrapartum care competency), McMullan et al. [24] (drug calculation ability), Bloomfield et al. [16] (handwashing skills), Sassen et al. [32] (shared decision making), and Kaveevivitchai et al. [21] (vital signs assessment skill). Our findings also provided evidence that flipped classrooms not only improve students’ clinical skills but also enhance their higher-level competencies (communication and teamwork capabilities, critical thinking, caring, work ethic, and accountability), which are required by ADN students who enter the RN-to-BSN program [1]. These results may have been driven by the advantages of the flipped classroom approach, which provides students with more peer communication, knowledge validation (during TBL and simulation activities), use of real clinical cases to engage student’s visually, and flexible access to materials [8, 20, 33].

### Evaluation of the differences between pre- and post-intervention scores on the MINS

To develop higher-level nursing competencies, such as critical thinking and analysis, the integration of evidence-based practice, problem-solving skills, etc., which are influenced by nursing students’ levels of comfort, confidence, and self-efficacy, is necessary [34, 35]. A person’s understanding of his or her own learning processes is known as metacognition [36], and this consists of awareness, cognitive strategy, planning, and self-checking, which are important for allowing nursing students to engage in clinical learning and problem solving [37, 38]. Our findings showed that our flipped classroom teaching approach could be a beneficial strategy for the development of metacognitive ability, and this method may enhance students’ higher-level nursing competencies and critical thinking, which was consistent with the findings of Hsu and Hsieh [39] and Jang and Hong [31]. In particular, the simulation was the preferred activity of the flipped classroom, which placed an emphasis on the direct translation of knowledge to practice rather than knowledge for its own sake [33, 40, 41].

Our study also demonstrated that those in the EG had significantly higher scores in the self-monitoring domain, which plays a crucial role in problem-solving. This indicates that students in the EG may have monitored their knowledge, which increased their awareness of effective skills for monitoring their progress toward obtaining solutions for the issues at hand [39]. However, the results regarding the self-awareness subdomain were the opposite, with the CG showing a significant improvement in self-awareness. This may have been because they recognized their limitations and changed their learning strategies to improve their adjustment to the ever-changing health care environment [29, 42].

### Evaluation of the differences between pre- and post-intervention scores on SDLRS

Self-directed learning, a central element in e-learning, has been widely used in professional health care disciplines; however, there is little or mixed evidence concerning its impact on learning outcomes [43, 44]. Our findings indicated that those in the EG showed a higher interaction pattern in terms of overall SDL scores, active learning, and love of learning than those in the CG. This differed from the findings of Gagnon et al. [43], indicating that BL has no direct impact on knowledge acquisition, satisfaction, or self-learning readiness. This result might come from increasing a student’s motivation toward a self-directed learning habit. Specifically, the active learning and love of learning sub-domains are two crucial constructs in nursing students’ life-long learning core competencies, and these skills may help them address clinical problems of varying difficulties in a wide range of situations.

### Flipped classroom satisfaction questionnaire

Our finding that the flipped classroom produced high satisfaction scores at the end of the semester is consistent with those of several studies [18, 21, 24, 25, 45]. We believe the flipped classroom teaching approach was highly accepted by students for two reasons. First, the advantages of an asynchronous e-learning portion, including its flexibility, convention, and the capacity for students to be self-paced, catered to different learning style as well and met individual educational needs. Second, the face-to-face portion provided more peer interaction and immediate support from teachers when questions arose [9, 33, 46, 47].

### Limitations and suggestions for future research

This study is not without limitations. First, students were self-report and not blinded to the purpose of the study and, given that both groups were administered the same program at the same institution, cross-contamination may have occurred. While there were no significant differences in age and gender, it is not possible to secure homogeneity for the factors influencing the results of this study in addition to age and gender. Thus, the generalizability of the findings is limited. Second, faculty members on both campuses could have communicated or shared their teaching strategies, which may have influenced the results. Third, the nursing faculty experienced challenges in terms of producing online teaching materials. Lastly, we did not monitor the students on whether they familiarized themselves with material via the e-campus platform, which is something to be considered in future research as it may have affected our results.

Regarding future studies, factors that may influence students’ learning outcomes, such as degree of motivation, learning style, and frequency and duration of accessing online resources, must be considered. Further, the application of a randomized clinical trial study design and rigorous control for heterogeneity factors during the intervention period may confirm the flipped classroom’s probable effects.

## Conclusions

This study showed that a flipped classroom combined with hybrid teaching methods could be an effective learning strategy for an RN-to-BSN program. The students in this program were satisfied the flipped classroom teaching and demonstrated improvements in core competencies, metacognitive abilities, and self-directed learning. Thus, we feel the flipped classroom approach is one of the most suitable teaching methods for today’s complex revolution in nursing curricula, and may enhance nursing students’ abilities to address numerous challenges.

## Data Availability

The datasets used and/or analysed during the current study are available from the corresponding author on reasonable request.

## Abbreviations

RN: Registered nurse
ADN: Associate Degree in Nursing
BSN: Bachelor of Science in Nursing
PBL: Problem-based learning
TBL: Team-based learning
BL: Blended learning
L campus: Linkou
C campus: Chiayi
EG: Experimental group
CG: Control group
SECC: Self-Evaluated Core Competencies Scale
MINS: Metacognitive Inventory for Nursing Students
SDLRS: Self-Directed Learning Readiness Scale
GEE: Generalized estimating equation
